# Effect of a Humanized Diet Profile on Colonization Efficiency and Gut Microbial Diversity in Human Flora-Associated Mice

**DOI:** 10.3389/fnut.2021.633738

**Published:** 2021-02-23

**Authors:** Sashuang Dong, BenHua Zeng, Ling Hu, Yuling Zhang, Jiaqi Xiong, Jing Deng, Liyan Huang, ZhenLin Liao, Jie Wang, Hong Wei, Xiang Fang

**Affiliations:** ^1^College of Food Science, South China Agricultural University, Guangzhou, China; ^2^Department of Laboratory Animal Science, College of Basic Medicine Science, Third Military Medical University, Chongqing, China; ^3^Precision Medicine Institute, The First Affiliated Hospital, Sun Yat-sen University, Guangzhou, China

**Keywords:** gut microbiota, humanized diet profile, intestinal flora disease, colonization efficiency, human flora-associated mice

## Abstract

Human flora-associated (HFA) mouse models allow us to design interventions for human disease research to test specific hypotheses and explore the complex commensal microbiome while avoiding the ethical limitations of using humans as models to directly study intestinal flora diseases. However, few studies have investigated the effect of a humanized diet profile (coarse-feed diet; CFD) on colonization efficiency and gut microbial diversity in HFA mice. We tested the colonization efficiency and gut microbial diversity in germ-free Kunming (KM) mice fed a CFD or a purified feed diet (PFD) at 1, 2, and 4 weeks. Although the colonization efficiencies differed significantly (67.50–70.00% vs. 72.69–85.96%) in the HFA mice, the colonization efficiency of the PFD-fed HFA mice (85.96%) was significantly higher than that of the CFD-fed mice (69.61%) at 2 weeks. At 4 weeks, the colonization efficiency of the PFD-fed mice (72.69%) was comparable to that of the CFD-fed mice (70.00%). Additionally, the gut microbial diversity of the CFD-fed HFA mice was similar to that of a human fecal donor. Regarding the Kyoto Encyclopedia of Genes and Genomes colonic microbiota metabolic pathways, the CFD-fed HFA mice showed more similarities to the human donor than to the PFD-fed mice in amino sugar and nucleotide sugar metabolism, biosynthesis of amino acids, carbon metabolism, purine metabolism, and phosphotransferase systems. In conclusion, the humanized diet profiles of the CFD and PFD could help establish human microbiotas in mice. Constructing HFA mouse models fed a CFD for 4 weeks may be useful in researching human-derived intestinal diseases.

## Introduction

Owing to the development of high-throughput sequencing techniques and applications in mapping the human gut microbiome, using gnotobiotic animals to explore the complex commensal microbiome seems promising (1, 2). Animal models enable design interventions to test hypotheses regarding human diseases. Constructing animal models of the microbiota often includes “humanizing” the animal's microbiota by transplanting human microbiotas into germ-free animals (3). Humanized animals are necessary for understanding the complex commensal microbiome and its roles in health and disease (4).

The composition of the intestinal microbiota is driven by factors such as diet (5), antibiotic therapy (6), genotype, mode of giving birth (7, 8), age (9, 10), and antibiotics (11–13). Gut microorganismal colonization is more strongly affected by the environment than by host genetics (14). Nutrition, mainly controlled by diet, is a powerful environmental factor in shaping the microbial community composition (15). Gut microbiota alterations due to dietary changes have been extensively studied (16), and gut microbiotas change rapidly when diets are diversified.

Because of sustainable developments in food science and technology, consuming finely processed food has resulted in poor health in some people (17). As people are beginning to understand how diet influences the structure and activity of the intestinal flora, more attention is being paid to the roles of coarse grains in humanized diet profiles, including the various dietary fibers needed by many beneficial intestinal bacteria (18). Consumption of fermentable dietary fibers reshapes the gut microbiota structure and function (19). Human flora-associated (HFA) mice who were switched from a coarse-grain diet to a purified diet displayed rapid and significant microbiome changes in response to the dietary changes (20). However, HFA mice who were fed a purified feed diet (PFD) after colonization rather than the coarse-feed diet (CFD) showed differences that may have been related to the human volunteers or to the mouse diets. In our previous work, we used an HFA C57BL/6J mouse model to study the co-action of a high-fat diet and tea polyphenols on the gut microbiota and lipid metabolism (21); limited differences between the compositions and functions of the human and mouse microbiotas were noted in some cases (21, 22). Research has shown that short-term changes in dietary patterns may have no significant influence on the gut microbiota, whereas long-term diets can substantially affect the gut microbiota; for example, high-fat diets can change the gut microbiota compositions of obese patients (5, 23). The gut microbiota is also involved in metabolism. For example, several gut microorganisms modulate lipid accumulation, lipopolysaccharide content, and short-chain fatty acid production, thus affecting food intake (24).

**Figure 1:**
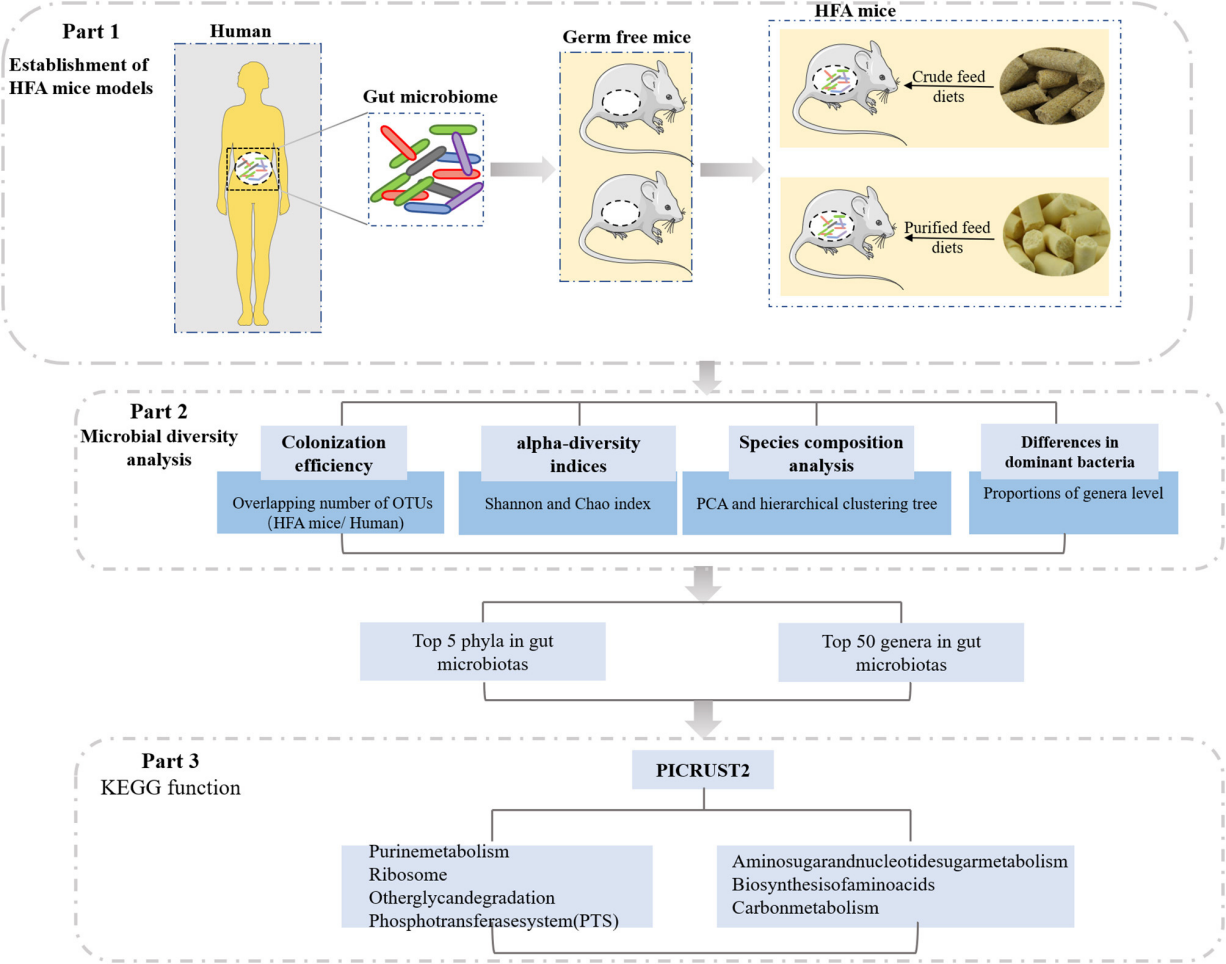
Study design.

In this study, we investigated the effects of different diets on HFA mice, which were constructed from germ-free mice by transfusing a human fecal suspension into their guts (Figure 1). The fecal bacteria from the mice inoculated with the human fecal suspension were similar to the fecal bacteria of human origin, and the stability of the offspring of the HFA mice suggested that the human microbes colonizing the mouse intestines had adapted to the mouse intestines. Therefore, we investigated the gradual adaptation of the fecal microbiota through a 4-week dietary intervention and analyzed it via 16S rRNA sequencing. Our data suggest that different dietary nutrients greatly affected the microbial colonization in these mice and that when constructing HFA mouse models, scholars should focus on interrelations in the diet to yield more relevant and personalized animal models.

## Materials and Methods

### Animals, Diets, and Experimental Design

Twelve 3–4-week-old male germ-free KM mice were obtained from the Department of Laboratory Animal Science, College of Basic Medical Sciences, Third Military Medical University, Chongqing, China. When the experiment began, all animals were housed in a sterile environment and were raised in sterile Trexler-type plastic film isolators (Feng Shi Laboratory Animal Equipment, Suzhou, China) at 23 ± 2°C under 40–70% humidity and a 12-h/12-h light/dark cycle. The feces and skin of the germ-free mice were microbiologically tested according to Chinese Laboratory Animal Microbiological Standards and Monitoring (GB-T 14926A-2001) to ensure the animals were germ-free before feeding.

The CFD (D94811508) and PFD (AIN-93G) diets were provided by the Laboratory Animal Center of Third Military Medical University and sterilized with 50 kGy of Co-60 (4Mrad, Radiation Center of Third Military Medical University). Supplementary Table 1 lists the ingredients in both diets. The water, bottles, and bedding were sterilized with high-pressure steam at 121°C and 110 KPa for 30 min. The 12 germ-free KM mice were randomly divided into the CFD group (*n* = 6, fed the CFD during all experiments) or the PFD group (*n* = 6, fed the PFD during all experiments).

### Establishment of HFA Mice Models

Fresh feces were collected from a healthy 25-year-old female college student who typically ate three meals daily at the university cafeteria. The healthy volunteer ate both vegetarian and meat diets and was recruited from the Third Military Medical University. She adhered to a normal schedule and lifestyle and took no antibiotics for at least 3 months before providing the stool sample. She was clinically screened for infectious risk factors and microbiome-mediated diseases and underwent blood and stool screening in keeping with consensus guidelines on donor screening and stool testing (25). The donor feces were stored in a sterilized sealable 10 ml centrifuge tube and immediately sent to the laboratory and stored at −80°C. The feces (10 g) were resuspended in 90 ml of sterile 0.1 M phosphate-buffered saline (pH 7.2) under anaerobic conditions. All mice (*n* = 12) were inoculated by oral gavage (0.3 ml/mouse) with the human fecal suspension to establish the HFA mice.

### Sample Collection and DNA Extraction

Fecal samples were collected from the HFA mice at 1, 2, and 4 weeks after inoculation and immediately frozen at −80°C. Microbial community genomic DNA was extracted from the collected feces using the E.Z.N.A.^®^ soil DNA stool Mini Kit (Qiagen, CA, USA) according to the kit's instructions. The DNA extract was checked on 1% agarose gel, and the DNA concentration and purity were determined using a Nanodrop 2000 (Thermo Scientific, USA).

### 16S rRNA Gene Sequencing

The bacterial 16S rRNA gene, including the hypervariable V3–V4 regions, was amplified via PCR using the primer pairs: 338F (5′-ACTCCTACGGGAGGCAGCA-3′) and 806R (5′-GGACTACHVG GGTWTCTAAT-3′). Purified PCR products were pooled in equimolar and paired-end sequenced on the Illumina MiSeq platform (Illumina, San Diego, CA, USA). The raw reads were deposited into the NCBI Sequence Read Archive database (accession number: PRJNA644474).

### Gut Microbiota Analysis

Alpha diversity analyses, including a rarefaction curve analysis at 97% similarity and Chao and Shannon index analyses, were conducted using Mothur software (v.1.30.1). Based on the weighted UniFrac distance, a hierarchical clustering tree at the operational taxonomic unit (OTU) level was constructed and analyzed for all samples. A scatterplot of the principal component analysis (PCA) scores showed that closer group scores meant more similar species compositions in the groups. ANOSIM analysis using QIIME showed that the between-group differences were greater than the within-group differences. Bacterial compositions of the different communities at the phylum and genus levels were compared, and the different species were analyzed. Colonization efficiency was calculated by counting the overlapping numbers of OTUs between the recipient HFA mice and the human inoculates. The number of OTUs in the human inoculates were normalized to 100%, and the fraction was calculated for the HFA mice.

### Metabolic Function Prediction

Metabolic functions were predicted using PICRUSt software v23. The gene ID corresponding to each OTU was used to obtain the Kyoto Encyclopedia of Genes and Genomes (KEGG) pathway. KEGG orthology (KO) information corresponding to each OTU and the abundance of each KO were calculated. Taxonomic data were input into the PICRUSt software package and filtered according to the Kruskal-Wallis H-test. The 16S copy number was then normalized, the molecular functions were predicted, and the final data were summarized to identify the KEGG pathways (26).

### Statistical Analysis

GraphPad Prism 8.3 (GraphPad Software, La Jolla, CA, USA) was used for all analyses and graphs. All scientific data are expressed as the mean ± standard deviation (SD) and were tested for normality using the Anderson-Darling test and for equal variances using the Brown-Forsythe test in SPSS (Version 23.0 for Windows, SPSS Inc., Chicago, IL, USA). Data not meeting the above parametric conditions were ranked before further statistical tests. Differences in alpha diversity and multiple groups were determined using the Kruskal-Wallis test. Colonization efficiency results were analyzed via one-way analysis of variance (ANOVA) with Tukey's *post hoc* tests in SPSS with “diet” or “time” as factors. *P*-values < 0.05 and < 0.01 were considered statistically significant and highly statistically significant, respectively.

## Results

### The CFD Modulated the Overall Gut Microbiota Structure

**Figure 2:**
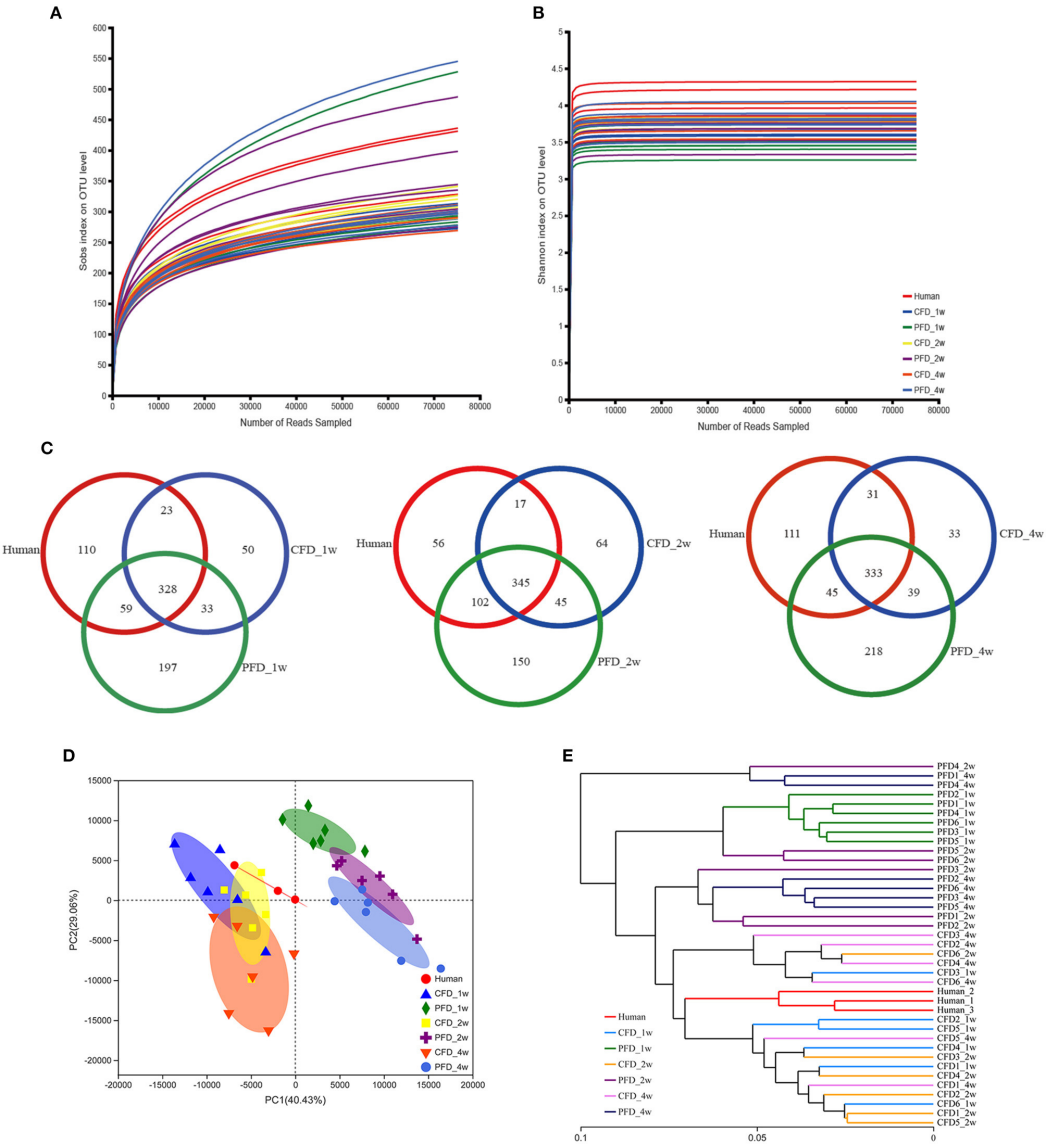
Overall profile of the gut microbiota. **(A)** Rarefaction curves of OTUs determined at the 97% similarity level. **(B)** Rarefaction curve according to the Shannon metric for the groups by the Mothur software. **(C)** Venn diagram of identified OTUs at 1, 2, 4 weeks. Sample sorting analysis. **(D)** Scatter plot of PCA-score shows the closer of the groups' points are, the more similar of the species composition in groups. Principal components (PCs) 1 and 2 explained 40.43 and 29.06%, respectively. **(E)** Based on the Weighted UniFrac distance, hierarchical clustering tree on the OTU level analysis is executed of all samples.

We sequenced 16S rRNA gene PCR products in the V3–V4 regions to explore the microbial community changes in the CFD-fed HFA mice. High-throughput sequencing generated 3,307,877 quality sequences from 39 samples, with an average of 84,817 ± 5,630 sequences per group. Gut bacterial community richness and diversity were determined in all samples by analyzing the Chao and Shannon indices. The rarefaction curves of the OTU numbers nearly achieved the asymptotes for all samples (Figure 2A), and the Shannon diversity index curves plateaued (Figure 2B). Colonization efficiency was measured by calculating the overlapping OTU numbers between the recipient HFA mice and the human inoculates. OTU overlapping revealed 328, 345, and 333 OTUs in the three groups at 1, 2, and 4 weeks, respectively (Figure 2C).

Colonization efficiency was evaluated by counting the overlapping numbers of OTUs between the recipient HFA mice and the human donor. The numbers of OTUs in the human inoculates were normalized to 100% (27), and the fractions were 67.50, 69.61, and 70.00% for the CFD-fed HFA mice and 74.42, 85.96, and 72.69% for the PFD-fed HFA mice at 1, 2, and 4 weeks, respectively. The PCA scatterplot showed that as the space between the group points narrowed, the species compositions in each group became more similar (Figure 2D). Principal components 1 and 2 explained 40.43 and 29.06% of the variations, respectively. Using the weighted UniFrac distance, a hierarchical clustering tree was constructed for OTU-level analysis on all samples. The results showed that the gut microbial community compositions were similar between the CFD-fed mice and the human donor (Figure 2E). The CFD- and PFD-fed mice for weeks 1, 2, and 4 also showed significant separations between each other and the human donor (Figures 2D,E). Additionally, the plots for the 2-week CFD-fed mice were similar to those of the human donor (Figure 2D), revealing similarities between the microbiotas of these samples. However, the plots for the PFD group were distinct from those of the other groups (Figure 2D).

**Figure 3:**
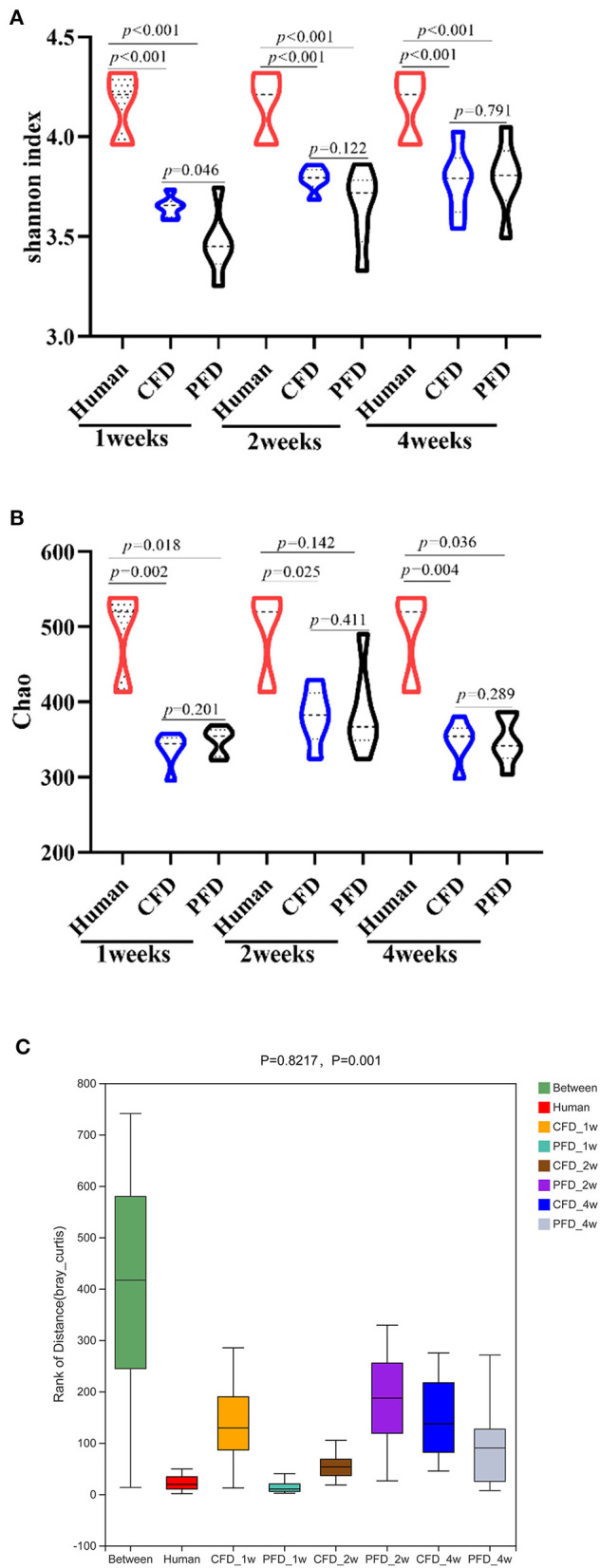
Bacterial alpha-diversity indices including **(A)** Shannon diversity index **(B)** Chao. **(C)** Distance calculated on OTU level of each sample group by Anosim analysis. There was a big difference in all groups. Statistical significance was denoted by *p* value.

Subsequently, we analyzed the gut microbiota diversity profiles between the CFD-fed and PFD-fed HFA mice. The Shannon and Chao indices revealed the diversity and richness, respectively, of the gut microbiotas (Figures 3A,B) and were highly significantly different between the human donor, the CFD mice, and the PFD mice at 1, 2, and 4 weeks. However, no significant differences were found between the groups at 2 and 4 weeks. ANOSIM analysis showed a significant difference compared with one group (*R* = 0.8217, *P* = 0.001) and a significant difference between two groups (Figure 3C).

### The Diets Reshaped the Gut Microbial Community Compositions

**Figure 4:**
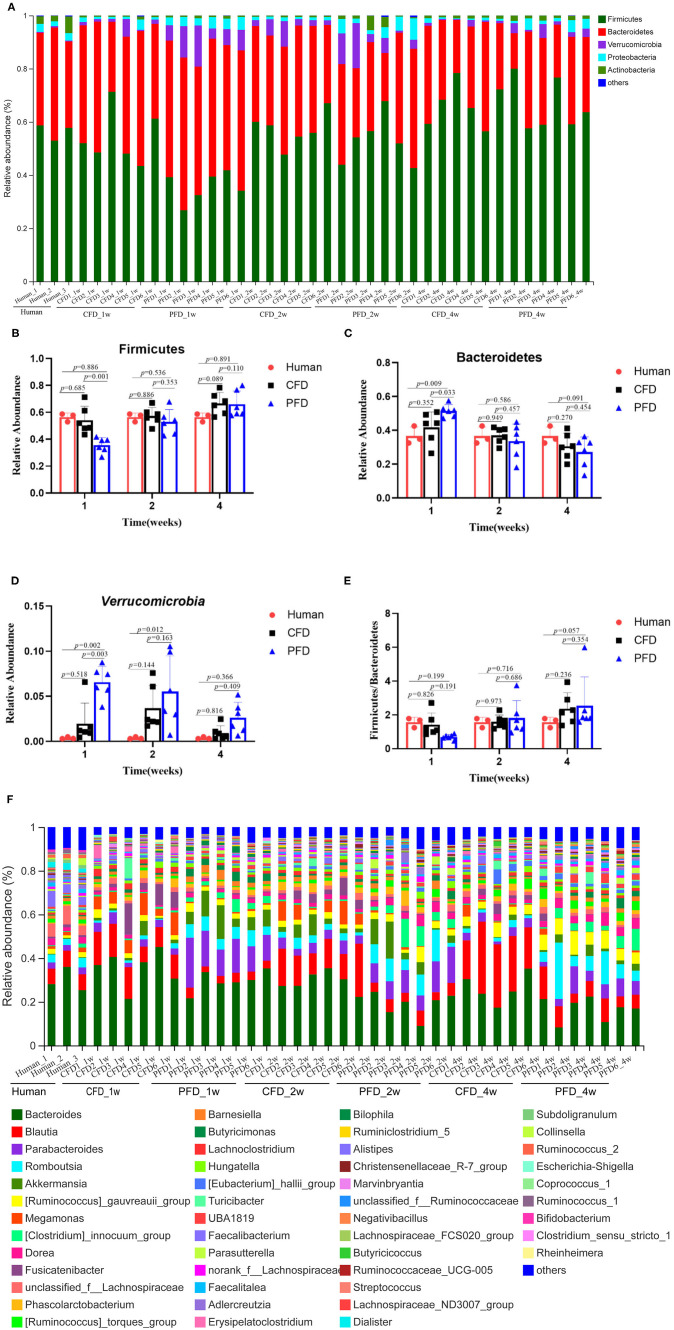
Relative abundance of gut microbiota. **(A)** Microbiota composition at the phyla level at 1, 2, and 4 weeks. Relative abundance for **(B)** Firmicutes, **(C)** Bacteroidetes, **(D)** Verrucomicrobia, **(E)** Ratio of Firmicutes to Bacteroidetes, **(F)** Microbiota composition at the genus level at 1, 2, and 4 weeks. Taxa with an abundance <1 % are included in “others”. The results were represented as mean ± SD. The statistical significance was determined using the One-way ANOVA and significant differences were indicated by *p*-value.

To understand the relative abundances of the microbial profiles, the phylum and genus-level compositions were analyzed for each group (Figures 4A,F). Among the classifiable sequences, five phyla were identified: Firmicutes, Bacteroidetes, Verrucomicrobia, Proteobacteria, and Actinobacteria, representing the dominant flora of the gut microbial communities in these samples (Figure 4A). The Verrucomicrobia abundance increased significantly, whereas Firmicutes and Bacteroidetes increased and decreased, respectively, at 1, 2, and 4 weeks (Figures 4B–D). Notably, the Firmicutes/Bacteroidetes ratio was increased significantly after treatment in both mouse groups at 2 and 4 weeks compared with that at 1 week (Figure 4E).

**Figure 5:**
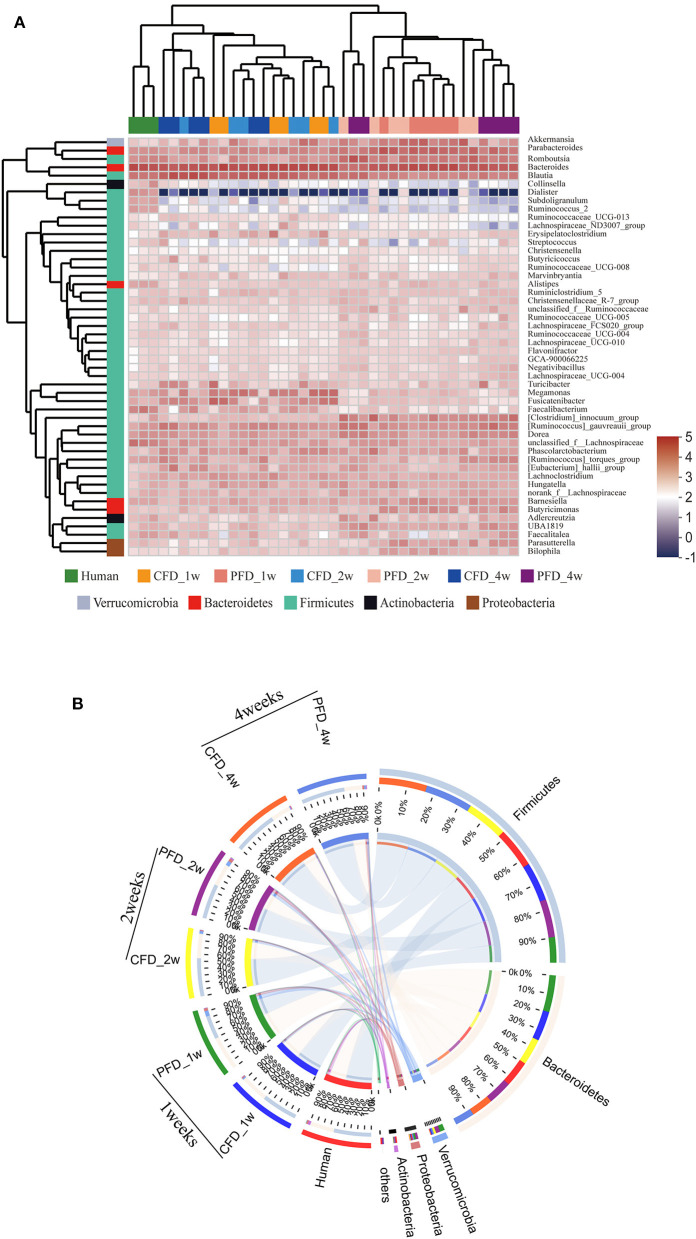
**(A)** Dendrograms of hierarchical cluster analysis at genus level and sample clustering are shown at left and bottom, respectively. Color gradient represents the datum values of relative abundances by l g. **(B)** The data were visualized by Circos. The width of the bars from each phylum indicate the relative abundance of that phylum in the group.

The dominant genera from the human donor, CFD-fed HFA mice and PFD-fed HFA mice belonged to Firmicutes and Bacteroidetes (Figure 5A). The predominant bacteria of the gut microbial communities from the CFD mice, PFD mice, and human donor were similar (Figure 5B). Data from Circos showed that Firmicutes and Bacteroidetes were present in the highest percentages among these three groups, at 56 and 37% for the human donor, 54 and 37% for the CFD mice, and 36 and 51% for the PFD mice at 1 week, respectively. Firmicutes increased from 57 to 67% and 53 and 68%, while Bacteroidetes decreased from 37 to 31% and 34 to 27% in the CFD-fed and PFD-fed mice at 2 and 4 weeks, respectively. The proportion of the dominant gut bacteria in the CFD-fed mice was similar to that of the human donor.

At the genus level, the sequences that were classified into the 50 dominant genera accounted for ~ 100% of the total sequences. *Bacteroides, Blautia, Parabacteroides, Romboutsia*, and *Akkermansia* represented the dominant genera in both mouse groups (Figure 4F), demonstrating that the gut microbial composition was reshaped after colonization in CFD-fed and PFD-fed HFA mice.

### The Dominant Taxa in the CFD-Fed HFA Mice Were Similar to those of the Human Donor

**Figure 6:**
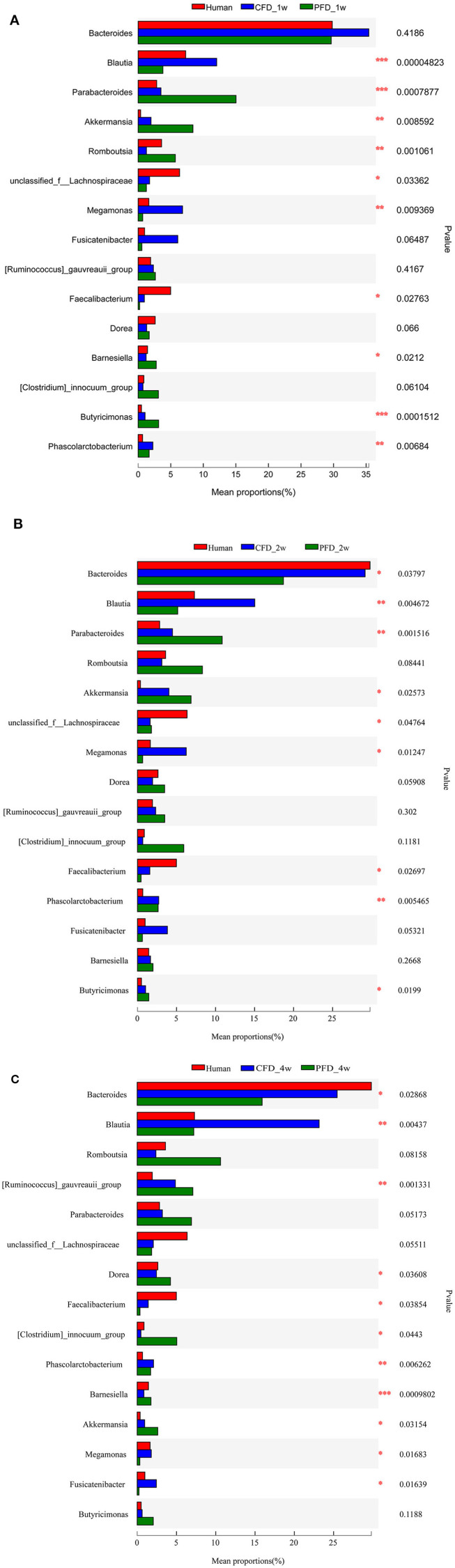
Mean proportions of the different genus at 1, 2, and 4 weeks. The one-way ANOVA test was used to evaluate the discrepancy of comparisons within three groups. ^*^p < 0.05, ^**^p < 0.01, ^***^p < 0.001.

We compared the mean proportions of the different genera at 1, 2, and 4 weeks for the human donor, CFD mice, and PFD mice (Figures 6A–C). At the genus level, *Bacteroides* was the most abundant in all three groups at 1 week and the most abundant in the human donor and CFD mice at 2 and 4 weeks. *Blautia, Akkermansia, Faecalibacterium, Phascolarctobacterium*, and *Megamonas* differed significantly among the three groups. Specifically, the *Blautia* abundance was significantly higher in the CFD mice than in the human isolate or PFD mice, and the discrepancy became more pronounced over time. Research has shown that *Blautia* is significantly reduced in obese individuals, especially in those who are insulin-resistant (28). Thus, we concluded that CFD-fed HFA mouse models would be healthier than PFD-fed mouse models.

### Functional Differences in Each Group

**Figure 7:**
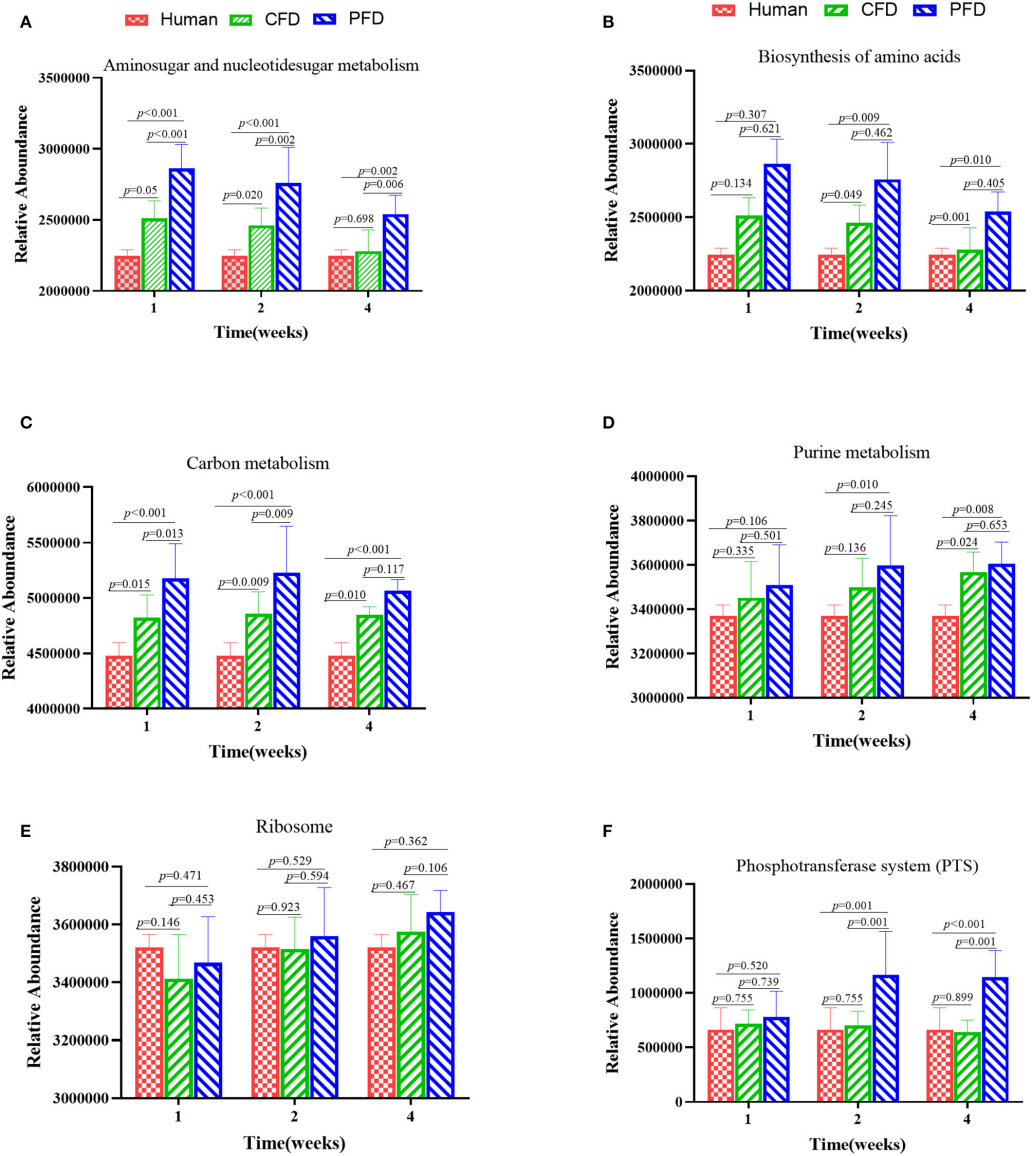
Altered metabolic pathways of the colonic microbiota among the groups in the KEGG database **(A–F)**. **(A)** Aminosugar and nucleotidesugar metabolism; **(B)** Biosynthesis of amino acids; **(C)** Carbon metabolish; **(D)** Purine metabolism; **(E)** Ribosome; **(F)** Phosphotransferase system (PTS). The results were showed as mean ± SD and filtered according to the Kruskal-Wallis H-test. Statistical significance was denoted by *p*-value.

To identify functional biomarkers of the KEGG pathways in each group, we performed a functional analysis to find differences among the three groups (Figure 7). Analyzing the KEGG pathway functions showed that amino sugar and nucleotide sugar metabolism, biosynthesis of amino acids, carbon metabolism, purine metabolism, ribosomes, and phosphotransferase systems (PTS) were the five highest functions. The results revealed that the metabolic pathways of the colonic microbiota, including amino sugar and nucleotide sugar metabolism, biosynthesis of amino acids, carbon metabolism, purine metabolism, and PTS in the CFD-fed mice were more similar to those of the human donor than to those of the PFD-fed mice. Thus, CFD-fed HFA mice may be better suited for research on human intestinal diseases.

## Discussion

The gut microbiota is the most extensively studied human-associated microbiota and plays a crucial role in human health. Several studies on the gut microbiota have shown that dietary factors can modulate the gut microbiota composition and functions, especially in obese patients (29). Intestinal microbes are also key modulators of immunity (30), metabolic diseases (24), cardiovascular diseases (31), and other diseases. Many studies have used fecal samples to investigate the gut microbiome compositions of specific-pathogen-free mice. HFA mice provide an excellent model *in vivo* for studying human intestinal microbes; these mice overcome the various uncertainties in human experiments, such as genetics, the environment, and dietary factors. However, little information exists on how diet affects colonization and gut microbial diversity in HFA mice. We tested whether different diets could improve colonization efficiency in HFA mice fed either a CFD or PFD. The colonization efficiency of the PFD-fed mice (85.96%) was significantly higher than that of the CFD-fed mice (69.61%) at 2 weeks, whereas the colonization efficiency of the PFD-fed mice (72.69%) was comparable to that of the CFD-fed HFA mice (70%) at 4 weeks. Further, the gut microbial diversity of the CFD-fed mice was similar to that of the human donor. Firmicutes and Bacteroidetes were the most abundant phyla in the human gut microbiota and often accounted for >90% of the total gut microbiota (32); the CFD- and PFD-fed mice showed comparable abundances. Unsurprisingly, different contents of ingredients such as protein (33), fat (34), fiber (19), digestible carbohydrates, and indigestible carbohydrates (35) in these two diets result in different abundances of dominant bacteria in mouse guts.

We used two different nutrient feeds to analyze their effects on gut microbial colonization. The protein content in the CFD was higher than that in the PFD, while the carbohydrates, especially fiber, were higher in the PFD. Firmicutes, Bacteroidetes, and Verrucomicrobia were the most dominant phyla in the samples. Verrucomicrobia was predominantly positively associated with fat and fiber but negatively associated with protein, whereas Firmicutes was positively associated with protein. High fat content can cause microbial dysbiosis characterized by a decreased relative abundance of Bacteroidetes and increased abundances of Firmicutes and Proteobacteria (36). Dietary fiber can stimulate the abundance of the resident bacterial. In our study, as the colonization time increased, the numbers of Bacteroidetes in the CFD mice were higher than those in the PFD mice, which is consistent with the increased Bacteroides in the intestines of people who have consumed long-term high-protein diets. At 1 week, the Bacteroidetes abundance was higher in the guts of the PFD mice than in the sample from the human donor but declined sharply at 2 and 4 weeks and reached a similar value to that of the human donor at 2 weeks. Firmicutes yielded a similar result, suggesting that the HFA mice had adjusted to the host microenvironment after 2 weeks. The mice grew similarly on both diets, indicating that the experimental diet did not induce major metabolic changes in the mice.

Our results suggest that diets can influence the gut microbiota (37, 38) and that the gut microbiota may be involved in diet-induced obesity (39). An extensive metagenomics study showed lower bacterial diversity and Bacteroidetes proportions but higher Actinobacterial proportions in obese than in lean individuals, while Firmicutes proportions did not differ significantly. Thus, the Firmicutes/Bacteroidetes relative abundance ratio could be deemed a biomarker indicative of obesity susceptibility (40). The intestinal microbiota mainly comprises Firmicutes and Bacteroidetes, and the Firmicutes/Bacteroidetes ratio can be used as a reference to determine the balance of intestinal bacteria (28). Increased Firmicutes/Bacteroidetes ratios in both humans and mice have been consistently associated with higher obesity and disease occurrence rates (41, 42). Furthermore, *Blautia* abundances also differ between obese and healthy individuals. *Blautia* spp. were significantly reduced in obese individuals and even more so in insulin-resistant obese individuals. Decreases in *Blautia* spp. in the intestinal flora of obese people are associated with intestinal inflammation and a deteriorated metabolic phenotype (43). At 1, 2, and 4 weeks, *Blautia* abundances in the CFD mice were significantly higher than those from the human donor and PFD mice. PFDs are richer in fiber and confer metabolic benefits on body weight and glucose control. Intestinal flora ferment dietary fiber to short-chain fatty acids. Butyrate can prevent high-fat diet-induced obesity and insulin resistance (44, 45), and propionate can prevent weight gain in obese people (46). Carbohydrate labeling revealed that acetate can accumulate in the hypothalamus, expressing an anorectic neuropeptide expression profile and resulting in mice failing to gain weight when consuming a high-fat diet supplemented with fiber (47). This indicated that propionate and butyrate activated the expression of gluconeogenic-related genes (48). Therefore, a PFD should be used to construct an obese HFA mouse model.

Finally, the intestinal microbiotas of the PFD mice were dominant in the functions of amino sugar and nucleotide sugar metabolism, biosynthesis of amino acids, carbon metabolism, purine metabolism, and PTS than were those of the CFD group. The changes in the gut microbiotas were always synchronous with function. Understanding the structure and function of intestinal bacteria may help prevent and treat some metabolic diseases. PFDs differ substantially from typical CFDs, which are often solely based on protein, fat, fiber, ash, calcium, and phosphorus. Additionally, CFDs often have higher energy contributions from protein and fat. Cases focusing on the interrelationships between diet and gut microbial ecology as well as in genetic animal contexts should yield more relevant and personalized animal models.

## Data Availability Statement

The datasets presented in this study can be found in online repositories. The names of the repository/repositories and accession number(s) can be found at: https://www.ncbi.nlm.nih.gov/, PRJNA644474.

## Ethics Statement

The animal study was reviewed and approved by Laboratory Animal Center, College of Basic Medicine Science, Third Military Medical University.

## Author Contributions

XF and HW conceived the research framework. LHu performed the experiment. SSD prepared the drafting of manuscript, analysis, and interpretation of data. Y-LZ participated in the writing of part of the manuscripts. J-QX upload sequence to NCBI. JD and L-YH assisted in organizing experimental samples. B-HZ, JW, and Z-LL revised the initial manuscript critically. All authors contributed to manuscript revision and read and approved the submitted version.

## Conflict of Interest

The authors declare that the research was conducted in the absence of any commercial or financial relationships that could be construed as a potential conflict of interest.
