# Corrigendum: Effect of a Humanized Diet Profile on Colonization Efficiency and Gut Microbial Diversity in Human Flora-Associated Mice

**DOI:** 10.3389/fnut.2021.676085

**Published:** 2021-04-26

**Authors:** Sashuang Dong, BenHua Zeng, Ling Hu, Yuling Zhang, Jiaqi Xiong, Jing Deng, Liyan Huang, ZhenLin Liao, Jie Wang, Hong Wei, Xiang Fang

**Affiliations:** ^1^College of Food Science, South China Agricultural University, Guangzhou, China; ^2^Department of Laboratory Animal Science, College of Basic Medicine Science, Third Military Medical University, Chongqing, China; ^3^Precision Medicine Institute, The First Affiliated Hospital, Sun Yat-sen University, Guangzhou, China

**Keywords:** gut microbiota, humanized diet profile, intestinal flora disease, colonization efficiency, human flora-associated mice

In the original article, there was a mistake in the legend for ^**^Figure 4^**^ as published. ^**^Legends of Figure B and C are missing^**^. The correct legend appears below.

**Figure 4:**
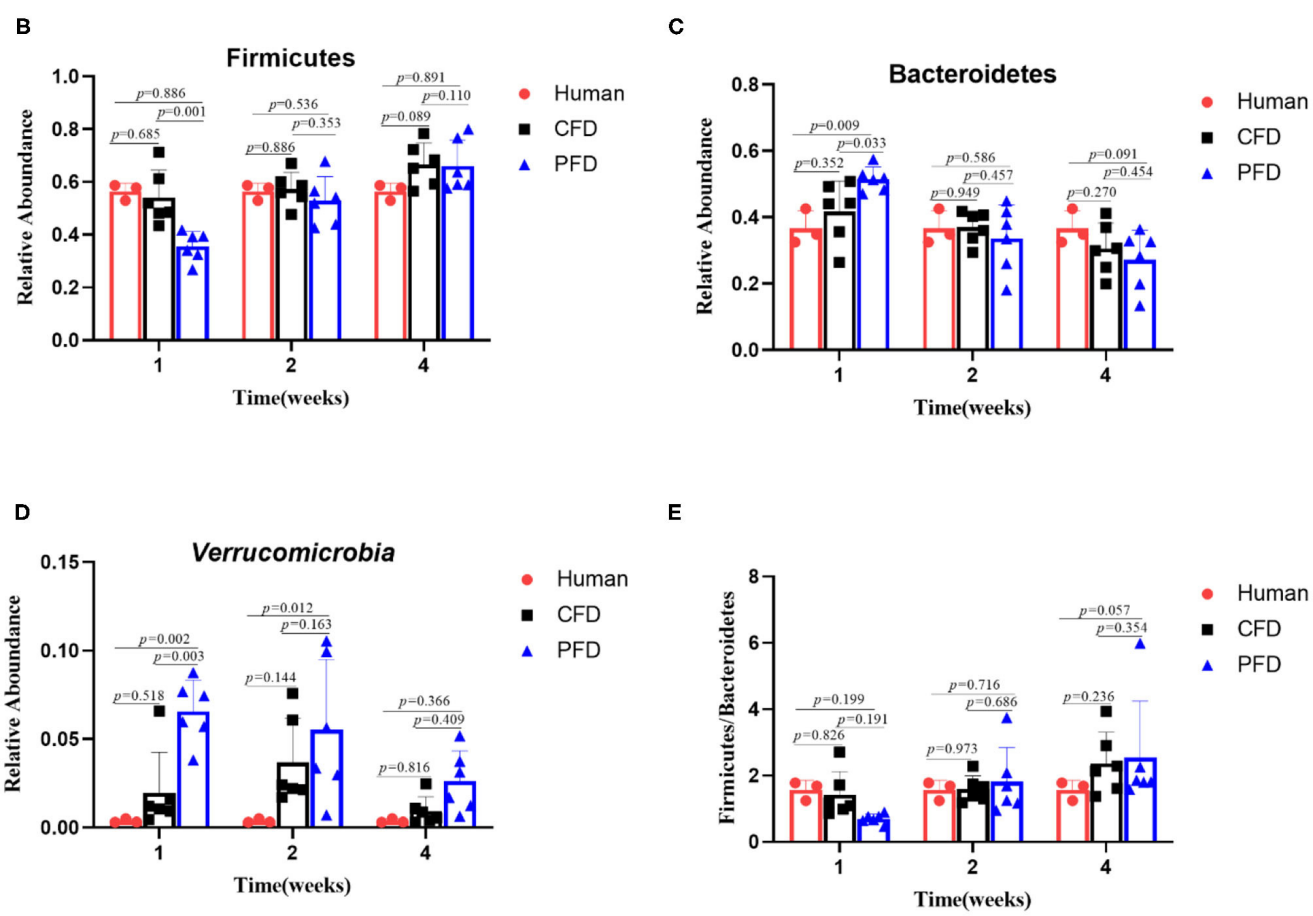
Relative abundance of gut microbiota. Relative abundance for **(B)** Firmicutes, **(C)** Bacteroidetes, **(D)** Verrucomicrobia, **(E)** Ratio of Firmicutes to Bacteroidetes.

The authors apologize for this error and state that this does not change the scientific conclusions of the article in any way. The original article has been updated.

