# Supplementary material for: Effect of a Humanized Diet Profile on Colonization Efficiency and Gut Microbial Diversity in Human Flora-Associated Mice
Source: Front Nutr. 2021 Feb 23;8:633738. doi: 10.3389/fnut.2021.633738 (PMC7940529; doi:10.3389/fnut.2021.633738)
Supplement: Supplementary file 1 [file Data_Sheet_1.docx]

**A**

| Ingredient | Among/kg  mixed diet |
| --- | --- |
| Cornstarch | 397.486 |
| Casein (≥85% protein) | 200.000 |
| Dextrinized cornstarch  (90-94% tetrasaccharides) | 132.000 |
| Sucrose | 100.000 |
| Soybean oil (no additives) | 70.000 |
| Fiber | 50.000 |
| Minera mix (AIN-93G-MX) | 35.000 |
| Vitamin mix (AIN-93-VX) | 10.000 |
| L-Cystine | 3.000 |
| Choline bitartrate (41.1 % choline) | 2.500 |
| Tert-butylhydroquinone | 0.014 |

| Ingredient | Among/kg  mixed diet |
| --- | --- |
| Moisture | 97.4 |
| Protein | 221.0 |
| Fat | 55.0 |
| Fiber | 44.0 |
| Ash | 65.0 |
| Ca | 11.9 |
| P | 7.6 |

**B**
